# Multicenter intracranial EEG dataset for classification of graphoelements and artifactual signals

**DOI:** 10.1038/s41597-020-0532-5

**Published:** 2020-06-16

**Authors:** Petr Nejedly, Vaclav Kremen, Vladimir Sladky, Jan Cimbalnik, Petr Klimes, Filip Plesinger, Filip Mivalt, Vojtech Travnicek, Ivo Viscor, Martin Pail, Josef Halamek, Benjamin H. Brinkmann, Milan Brazdil, Pavel Jurak, Gregory Worrell

**Affiliations:** 1 Mayo Systems Electrophysiology Laboratory, Department of Neurology, Mayo Clinic, Rochester, MN USA; 2 Brno Epilepsy Center, Department of Neurology, St. Anne’s University Hospital and Medical Faculty of Masaryk University, Brno, Czech Republic; 3 The Czech Academy of Sciences, Institute of Scientific Instruments, Brno, Czech Republic; 4 Department of Physiology and Biomedical Engineering, Mayo Clinic, Rochester, USA; 5 Czech Institute of Informatics, Robotics, and Cybernetics, Czech Technical University in Prague, Prague, Czech Republic; 6 International Clinical Research Center, St. Anne’s University Hospital Brno, Brno, Czech Republic; 7 CEITEC – Central European Institute of Technology, Masaryk University, Brno, Czech Republic

**Keywords:** Epilepsy, Data mining

## Abstract

EEG signal processing is a fundamental method for neurophysiology research and clinical neurology practice. Historically the classification of EEG into physiological, pathological, or artifacts has been performed by expert visual review of the recordings. However, the size of EEG data recordings is rapidly increasing with a trend for higher channel counts, greater sampling frequency, and longer recording duration and complete reliance on visual data review is not sustainable. In this study, we publicly share annotated intracranial EEG data clips from two institutions: Mayo Clinic, MN, USA and St. Anne’s University Hospital Brno, Czech Republic. The dataset contains intracranial EEG that are labeled into three groups: physiological activity, pathological/epileptic activity, and artifactual signals. The dataset published here should support and facilitate training of generalized machine learning and digital signal processing methods for intracranial EEG and promote research reproducibility. Along with the data, we also propose a statistical method that is recommended for comparison of candidate classifier performance utilizing out-of-institution/out-of-patient testing.

## Abstract (summary)

**Table:** 

Measurement(s)	brain measurement • physiological activity • epileptic seizure AE • Artifact • Annotation
Technology Type(s)	electroencephalography (EEG) • intracranial electroencephalography • data transformation
Factor Type(s)	institution
Sample Characteristic - Organism	Homo sapiens

Machine-accessible metadata file describing the reported data: 10.6084/m9.figshare.12326471

## Background & Summary

Intracranial electroencephalography (iEEG) is an invasive procedure commonly used for localization of epileptic seizure onset zones in patients with drug resistant epilepsy. The iEEG signals are directly measured from cortical and deep brain structures, e.g. hippocampus, amygdala, etc. Currently, the visual inspection and artifact rejection of the data is standard pre-processing procedure that must be done prior to evaluating of epileptic seizure onset zones. The improvement of EEG acquisition systems, data storage, and surgical techniques allows for large scale data collection spanning over multiple days to weeks, recording from hundreds of electrodes with sampling rates reaching up to 32 kHz in research settings^1^. In addition to the clinical utility for mapping epileptic brain by localization of seizures and interictal epileptiform transients these data enable a wide range of neuroscience research activities. The amount of collected data is rapidly increasing and advancement in data compression, storage, visualization, and automated processing of data is important and has received significant attention^2,3^. The acquisition of large datasets has driven the development of improved data preprocessing tools that enable extraction of important application specific data segments i.e. to focus on the data that are clinically important like seizures or other brain states for research purposes. It is very burdensome for a human operator to manually classify hundreds of channels for data spanning days to weeks. If reliable automated methods, can be developed they can easily and reliably mine the data and crop the segments of the data with the iEEG features of interests. The automatic classification of artifacts and segmentation of iEEG recordings is recognized as a challenging task, and many interesting studies have been published addressing the challenges^4,5^. In recent years, a variety of methods using machine learning techniques and deep learning techniques for iEEG processing emerged with impressive results^6–8^. The generalizability of an automated artifact detection method will enable broader utilization and extension for any retrospective and prospective iEEG dataset, but this has received little attention. We have recently demonstrated robust generalization of automated detection algorithms for artifact classification using training and testing datasets collected from different institutions, acquisition systems, under different measurement conditions^7,8^.

Automated processing and data mining with Convolutional Neural Networks (CNN) are powerful, but the interpretation of particular classifications and correlation with known iEEG waveforms is difficult. The inability to dissect the CNN decision process makes it less interesting for understanding fundamental neurophysiology, and ultimately for usage in clinical practice. We recently demonstrated the ability to temporally localize graphoelements that drive the final classification and make visual review and interpretation of raw EEG recordings possible^8^. This approach could also prove useful for supervised adaptive retraining in active learning and expert-in-the-loop scenario based on expert’s review of the data yielding false positive or false negative classifications. Common iEEG graphoelements of physiological activity (e.g. delta, alpha & beta bands oscillations) can be identified and characterized. Further, data contaminated with artifactual signals from several types including artificial or physiological sources. The most common artifact is powerline noise (50 Hz or 60 Hz) that is usually induced to the acquisition systems. Other artifacts have originally received less attention or were incorrectly assumed not to contaminate iEEG. But, it was later proven that eye movements and muscle artifacts might distort iEEG recordings^9–11^ and need to be either removed or discarded from analysis. In many recent electrophysiological studies across several domains (neurology, cognition, etc.), the research focuses more and more on subtle attributes of the iEEG signal such as power in high frequency bands^12–15^. In such analyses, it is critical to recognize and control for subtle power changes in the signal that might be caused for example by high frequency harmonics of power line noise and other artificial generators that would be previously omitted by cropping a frequency band (e.g. analyzing data in low frequencies only). In general, iEEG often contains artifactual signals in electrodes that are spatially closer to scalp or cranial nerve foramen (movement, muscle artifacts, eye movements). The ECG signal and weak scalp signals might also propagate to the measurement system by a common reference. In addition, natural pulsation of the brain tissue driven by respiration, cerebro-spinal fluid pulsation, and hearth rhythm (blood-flow and pulsation in vessels) might cause motion distortion artifacts. The patients undergoing the iEEG monitoring have electrodes implanted into the brain structures that are assumed to generate the epileptic/pathological activity like interictal epileptiform spikes and high-frequency-oscillations^13^. The problematic part of automated iEEG classification, that biases results of the studies, is the fact that artifactual signals (like muscle artifacts) caused by patient movement or other physiological sources commonly share features with pathological signals, e.g. power in band 200–600 Hz.

The purpose for public sharing of this dataset is to advance the field and the progress of generalized machine learning and iEEG processing techniques in neurophysiology. In particular, machine learning techniques capable of processing data from multiple institutions without performance degradation and without the need of retraining will be extremely useful. We anticipate these methods will boost the creation of new, large gold standard datasets from multiple institutions. The generalized pre-trained models should be re-trainable (transfer-learning)^16,17^ to adapt to new datasets without requiring a collection of new annotated gold standards, which should significantly decrease the time for manual annotation and therefore advance the iEEG utilization in clinical practice and research. For this reason, we believe that public sharing of such datasets is a cornerstone for further advancing iEEG research.

## Methods

### Data collection

The iEEG dataset published in this study was collected from two institutions: St. Anne’s University Hospital (Brno, Czech Republic) and Mayo Clinic (Rochester, Minnesota, United States of America). The data acquisition methods, and signal annotation techniques described below are adopted and expanded version of descriptions in our related work^7,8^. Here, for purposes of data sharing, we significantly extended the datasets that were used in our previous studies^7,8^. We provided additional information that are clinically relevant and might extend usability of the dataset. Each data segment is described by clinical useful features: classification category (power line interference; high frequency noise; pathological activity; physiological activity), seizure onset zone (True, False), anatomical location, electrode type, reviewer identification number, patient number. Provided information allows for various statistical testing scenarios. The description of the format of data and meta-data is extensively commented in section Data Format Description.

The St. Anne’s University Hospital (FNUSA) dataset was made up of iEEG data collected in awake resting state from 14 patients diagnosed with drug resistant epilepsy (DRE) who underwent a standard pre-surgical monitoring for localization of seizure onset zone, a standard for epilepsy surgery. The acquisition system used for the measurement in the hospital was a BrainScope system (M&I, BrainScope, Czech Republic). This system allows for recording up to 192-channel with maximum 25 kHz sampling rate and common reference montage. Here the system was used to record 30 minutes of awake resting interictal iEEG recordings with 25 kHz sampling rate. Raw data was filtered with 2 kHz low-pass filter, and down-sampled to 5 kHz to avoid aliasing. The electrodes used in all patients from the dataset were standard intracranial depth electrodes (5, 10 and 15 contact semi-flexible multi-contact platinum electrodes (ALCIS - Temis Health, France), with a diameter of 0.8 mm, a contact length of 2 mm, contact surface area 5.02 mm^2^ and inter-contact distance 1.5 mm).

**Fig. 1:**
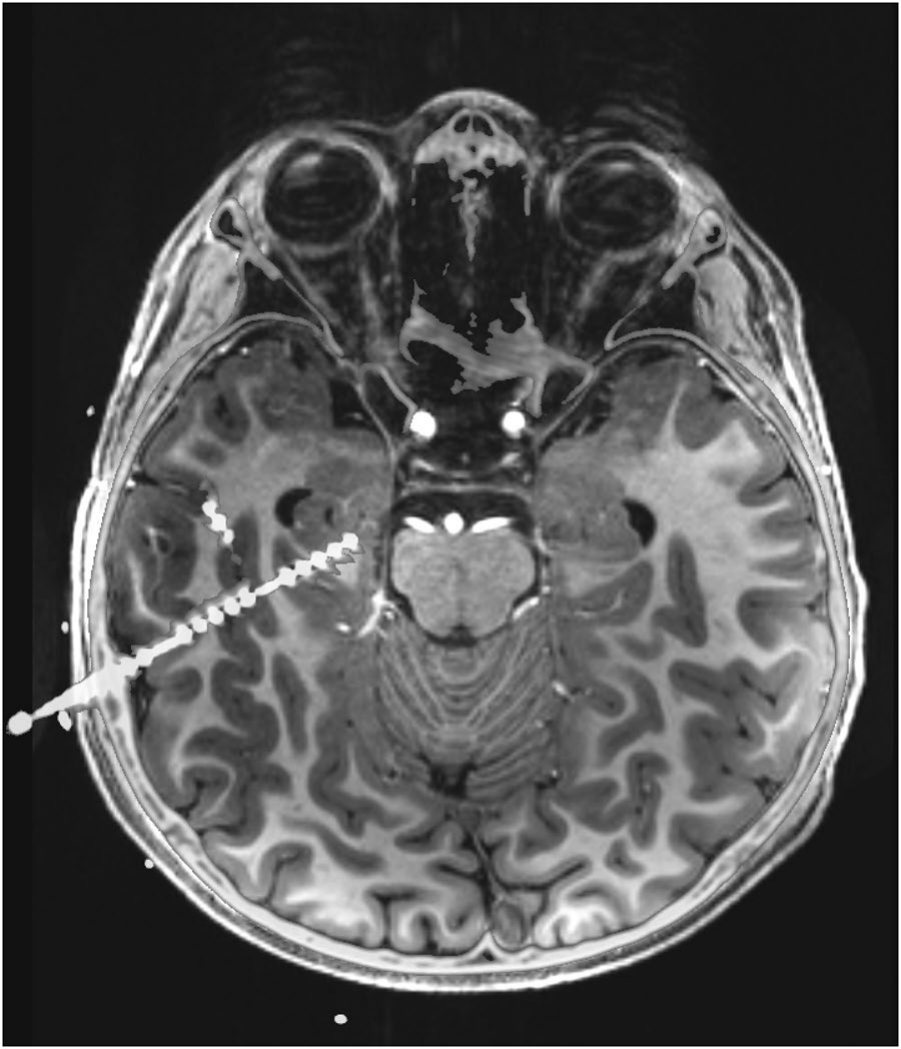
A patient undergoing invasive EEG monitoring with stereotactic depth electrodes had a high-resolution spiral CT image acquired following electrode placement for verification and to rule out hemorrhage. The CT images were co-registered to the patient’s pre-operative 7-Tesla T1 weighted MRI using MIM version 6.8.3 (Mim Software Inc.), with pixel intensities averaged to optimize concurrent visibility of CT electrodes and MRI tissue contrast.

Mayo Clinic data were recorded during the first night after electrode implantation and consisted of two-hour long iEEG recordings. The data were collected between 1 AM and 3 AM from 25 patients with DRE undergoing evaluation for epilepsy surgery. The Neuralynx Cheetah system (Neuralynx Inc., Bozeman MT, USA) was used to acquire the data at sampling rate of 32 kHz with hardware filter bandwidth of DC – 9 kHz. Similar to St’ Anne’s recordings, all data were filtered by an antialiasing filter, but in this case with cutoff frequency of 1 kHz. The data was subsequently down-sampled to 5 kHz. Patients were implanted with either depth electrodes or grids and strips, or the combination. An illustrative example (Fig. 1) shows co-registered electrode placement of fused MRI and CT scans of patient undergoing invasive EEG monitoring with stereotactic depth electrode. The depth electrodes used in the dataset were AD-Tech electrodes (AD-Tech Medical Instrument Corp., Racine, WI or PMT, Chahassen, MN, USA) and consisted of 4 or 8 Platinum/Iridium contacts (2.3 mm long, 1 mm diameter, spaced 5 or 10 mm center-to-center). AD-Tech subdural grids and strips electrodes had 4.0 mm diameter Platinum/Iridium discs (2.3 mm exposed) with 10 mm center-to-center distance.

### Ethics declaration

All subjects gave written informed consent in accordance with the Declaration of Helsinki. The protocol was approved by the Mayo Clinic Institutional Review Board and St. Anne’s University Hospital Research Ethics Committee and the Ethics Committee of Masaryk University.

### Data annotation

All data were reviewed in SignalPlant^18^, a free software tool for signal processing, inspection and annotation. The dataset was annotated by 3 reviewers, where each recording was reviewed by single reviewer. Each recording was visually reviewed in time domain alongside power distribution matrices (PDM) for manual artifact detection^13^. The PDM method estimates the signal power envelope by data filtering in specific frequency bands, and further computes the absolute value of the analytical signal (analytical signal is complex signal comprising original signal in the real domain and its Hilbert transform in imaginary domain) to obtain the signal envelope. Visual inspection of the PDM allow for fast localization of high-power events appearing across all channels and are likely to be artifacts. The signal regions with high power envelope were subsequently reviewed in the time domain to classify signals into pathological/artifactual group. Standard clinical evaluation of pathology of the brain substrate requires two-year post-surgical follow-up evaluating reduction of epileptic seizures. However, in this study, we are targeting identification of signal graphoelements and thus defining pathological signal group as signals with epileptiform graphoelements e.g.: HFOs and spikes or epileptiform discharges, that are visually reviewed and predominantly extracted from electrodes implanted to a brain structures like hippocampus. Generation of PDM is time consuming process, however, SignalPlant allows for CUDA GPU accelerated signal filtering^19^, which significantly speeds up the process. Annotated events were segmented with constant-length-segmentation into 3 sec (15000 samples) long data clips. The length of the constant-length-segmentation window was empirically estimated regarding the fact that muscle artifacts span over multiple seconds. This iEEG data window provides sufficient context to reliably differentiate between all classes of the data (physiological activity, pathological/epileptic activity, power-line noise, and other non-cerebral artifacts).

## Data Records

**Fig. 2:**
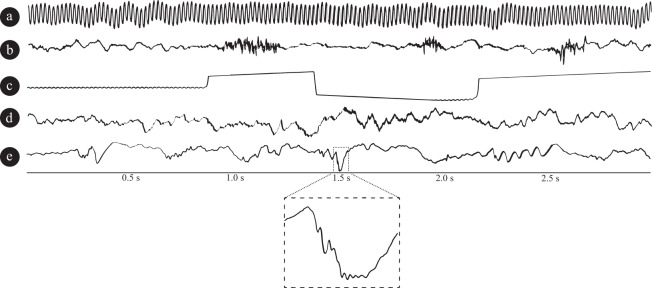
Figure shows intracranial EEG signal examples from different classification categories. (**a**) powerline noise, (**b**) muscle artifact, (**c**) baseline jump artifact, (**d**) physiological signal, and (**e**) epileptiform pathological signal with an HFO riding on a spike.

The datasets from St Anne’s University Hospital and Mayo Clinic consist of 155182 and 193118 data clips, respectively. The basic overview of segments distributions for each class/dataset is described in Table 1. Comprehensive data description statistics might be derived from datasets metadata files. In general, datasets contain data clips from four groups of distinctive events: powerline noise (in our case 50 Hz or 60 Hz depending on power line frequency at clinic’s location); muscle and machine artifacts; physiological iEEG activity in different behavioral states of subject (sleep/wake/wake-relax); pathophysiological activity. For example, Fig. 2a shows signals recorded in FNUSA contaminated by 50 Hz noise. Figure 2b shows movement artifact and Fig. 2c illustrates baseline jumps caused by instrumentation). Muscle, movement and machine artifacts group is iEEG recording that contains most often high frequency components caused either by movements, muscle artifactual activity of subject or artifacts caused by instrumentation. Figure 2d shows normal wake-relaxed state iEEG activity from FNUSA dataset. Pathological/epileptiform activity can consist from interictal epileptiform activity like spikes or high frequency oscillations (Fig. 2e). The datasets are publicly available to use under CC0 license and might be downloaded from figshare^20^ repository.

**Table 1:** St Anne’s University Hospital (FNUSA) and Mayo Clinic Datasets.

Classification category	St. Anne’s University Hospital (FNUSA)	Mayo Clinic
Physiological Activity	94560	56730
Pathological Activity	52470	15227
Artifacts	32599	41303
Power line noise (50 Hz/60 Hz)	13489	41922
Total	193118	155182

The table shows the number of 3-second examples for each classification category. The datasets described in this table are extended versions of previously used data in our related research^7,8^.

## Technical Validation

**Fig. 3:**
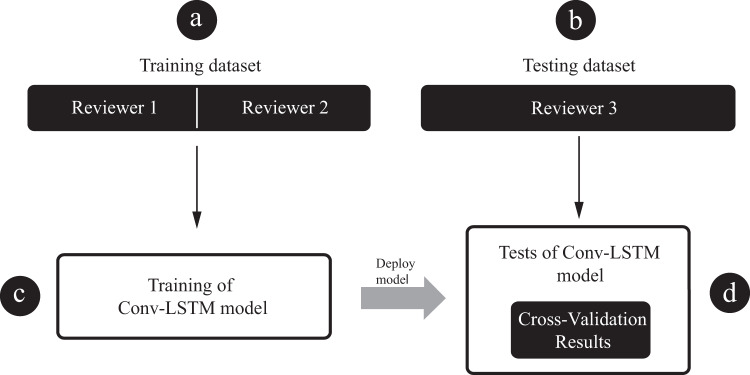
Diagram depicts pipeline used for cross-validation testing of generalized model. (**a**) training dataset, (**b**) testing dataset, (**c**) generalized model training, (**d**) testing phase.

**Fig. 4:**
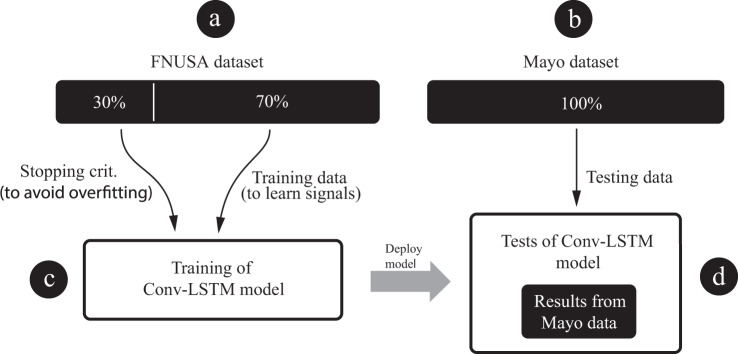
Diagram depicts pipeline used for out-of-institution testing of generalized model. (**a**) training dataset, (**b**) testing dataset, (**c**) generalized model training, (**d**) out-of-institution testing.

In order to validate the reliability of gold standard annotations, we have used a cross-validation statistics in predicting the class by model that has been trained by annotations from another reviewer. For example, model was trained on data classified by reviewers 1 and 2 and subsequently tested on out of sample data segments classified by reviewer 3 (Fig. 3, Table 2). This procedure was repeated for each reviewer. Moreover, we provide out-of-institution testing statistics in order to show that annotations are consistent across institutions (Fig. 4, Table 3). Given methods provides a measure of data labeling quality. In order to validate the annotations, we used the Convolutional LSTM neural network that was previously described^8^. The model processes z-score normalized spectrograms of data and provides probability for each classification group. Here, we used the standard metrics i.e.: area under the receiver operating characteristic (AUROC) and area under the precision-recall curve (AUPRC) that are commonly applied in evaluation of model classification performance. For class imbalance datasets, like in our case, it’s crucial and more objective to report both AUROC and AUPRC in order to show unbiased model performance.

**Table 2,:** Table describes cross-validation results for each reviewer separately.

Reviewer	Mayo Clinic		FNUSA	
	AUROC	AUPRC	AUROC	AUPRC
1	0.97	0.92	0.94	0.86
2	0.99	0.97	0.87	0.72
3	0.95	0.91	0.91	0.83
AVG	0.97	0.93	0.92	0.80

Standardly used classification metrics: AUROC and AUPRC are reported.

**Table 3:** Table describes out of institution testing. Standardly used classification metrics: AUROC and AUPRC are reported.

Training set	Testing set	AUROC	AUPRC
St. Anne’s (FNUSA)	Mayo Clinic	0.80	0.71
Mayo Clinic	St. Anne’s (FNUSA)	0.84	0.74

### Data format description

We publish this dataset in a format that allows easy accessibility to a general machine learning community and allows for optimal and fast machine learning. For this reason, the datasets are stored in two separate zip archives, where each archive consist of data records from one institution. Each data segment is saved in.mat file format in order to allow processing in commonly used computing tools like Matlab and Python. Proposed format was recognized as very easy to work with during several machine learning competitions for classification of electrophysiological signals (mostly from cardiology domain) e.g. Computing in Cardiology Challenges^21^. Data segments are saved as data vectors (1 × 15000 float vector). Each dataset contains coma separated value (segments.csv) document describing metadata for each segment i.e.: segment_id, channel, category, reviewer, seizure onset zone (SOZ), anatomy, electrode type, anonymized patient_id, and institution. At the same time, we published the datasets in iEEG-BIDS format to comply with neuro data sharing standard^22,23^. Datasets and annotations are stored in multi-scale electrophysiology file format^24^ (.mef) that is supported^23^ by BIDS^22^. An official C code libraries and documentation for.mef usage are publicly available at https://github.com/msel-source/meflib.

In order to promote data sharing and reproducibility of results, we also publish the example of the training code for neural network models along with the dataset. We also publish Python pipelines together with requirements for Python environment. This should allow for smooth data handling and help with using the dataset. Example codes are might be downloaded from figshare^20^ or github (https://github.com/xnejed07/NoiseDetectionCNN-GRU).

## Usage Notes

To streamline the data segmentation and machine learning process and to avoid an extra workflow on side of potential data users (direct manipulation with.mef files and compilation of supported C code libraries), we decided to publish the datasets that are segmented to 3-second segments (15,000 samples) also in matlab files (.mat). The 3-second length of the segment was empirically chosen based on electrophysiological characteristics of iEEG, experiments, tests, and results of our previous study^7^. Each segment is appropriately labeled to an assigned class with all the other meta-data provided. We encourage using this dataset for training of deep-learning methods for processing of new intracranial EEG data. The datasets might be used as a pretraining step. We assume that this will significantly increase the speed of automated annotation process of new data. We have previously published and described the transfer learning method^7^, that produces the probability matrices for each classification group. The organization of the dataset into small data clips allows for a rapid model development. That means that user doesn’t need to spend extensive amount of time with data annotation and other machine learning preprocessing steps, which usually consume a major part of time in development and testing of machine learning methods.
